# Massive Calcified Abdominal Aortic Aneurysm Presenting as Low Back Pain

**DOI:** 10.7759/cureus.46406

**Published:** 2023-10-03

**Authors:** Oxana Ushakova, Keyvan Ravakhah

**Affiliations:** 1 Internal Medicine, MetroHealth Medical Center, Cleveland, USA; 2 Internal Medicine, Mount Carmel Grove City, Grove City, USA

**Keywords:** abdominal aortic aneurysm, dementia vascular, major adverse cardiovascular event, lower back pain (lbp), calcified aneurysm

## Abstract

Calcified abdominal aortic aneurysm (CAAA) is a radiological finding that manifests the calcification in the bulged aortic walls. CAAA has high mortality. The presence of calcification as a key player in abdominal aortic aneurysm (AAA) rupture risk was reported in the literature. Factors contributing to a CAAA compared to AAA are age, dyslipidemia, hypertension, diabetes mellitus, genetics, disturbances in calcium-phosphate homeostasis, and smoking. There are a few genetic mutations associated with CAAA as well. Causes of AAA include lipid build-up in the aortic wall, inflammatory diseases, traumas, blood vessel diseases that supply the aortic wall, and connective tissue disorders.

## Introduction

Abdominal aortic aneurysm (AAA) is diagnosed when the abdominal aorta diameter is greater than 3 cm. At this stage, the patient undergoes a regular follow-up, and once the diameter exceeds 5.5 cm for men and 5 cm for women or rapidly expanding, who are asymptomatic, repair should be considered due to high risk of rupture. For symptomatic patients, repair can be done with any size of aneurysm. Calcified abdominal aortic aneurysm (CAAA) is frequently diagnosed incidentally and sometimes associated with specific gene expression leading to a significant calcium build-up [1]. CAAA has high mortality [2], and one study implied the presence of calcification as a key player in abdominal aortic aneurysm (AAA) rupture risk [3]. AAA is correlated with risk factors associated with an incorrect lifestyle, such as smoking, a wrong diet, absence of regular exercise, and gender [4]. Factors contributing to a CAAA compared to AAA are age, dyslipidemia, hypertension, diabetes mellitus, genetics, disturbances in calcium-phosphate homeostasis, and smoking [5]. There are a few genetic mutations associated with CAAA as well [6]. CAAA is a strong predictor of future cardiovascular events [7]. One of the studies reported an association between CAAA and late-life dementia which potentially might be used as a screening test [6].

## Case presentation

**Figure 1:**
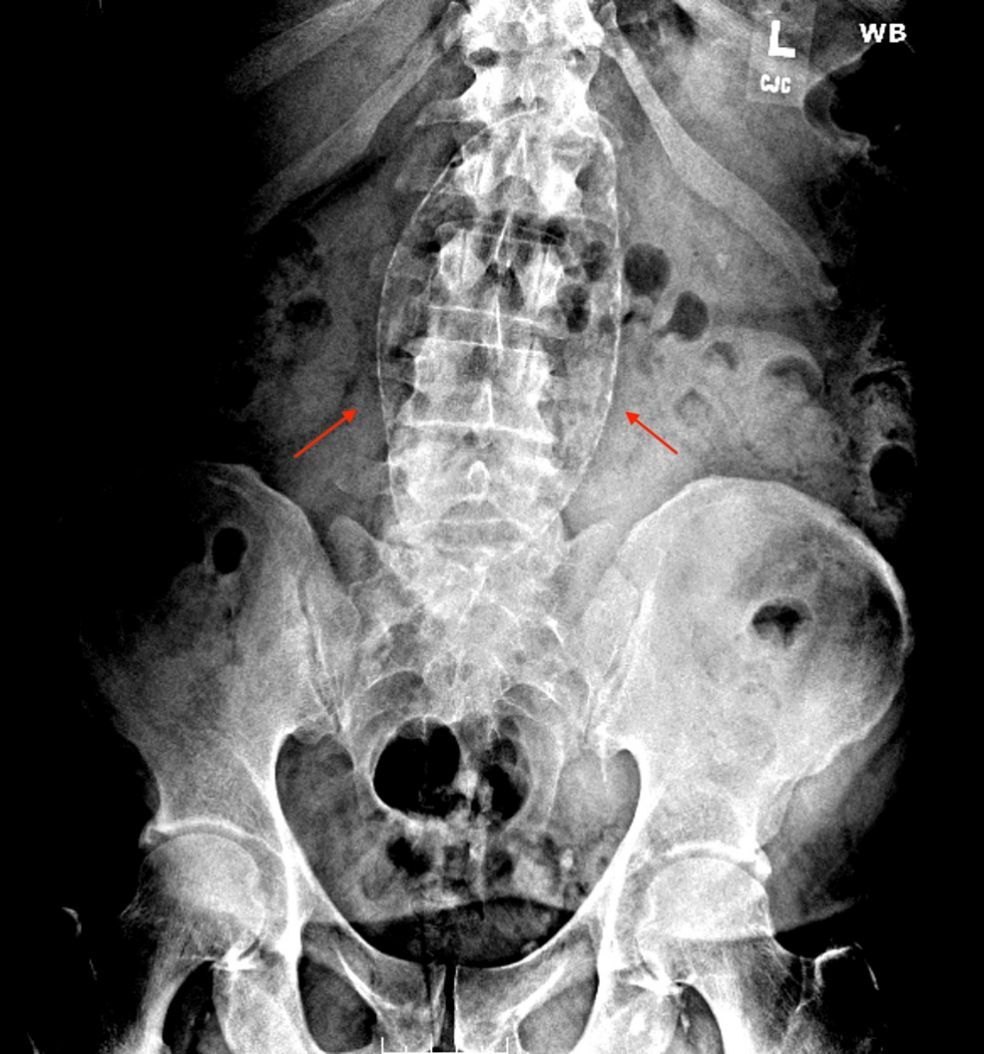
Calcified abdominal aortic aneurysm The abdominal aortic aneurysm sized 7.4 cm by 14.8 cm with distinct calcifications (pointed with red arrows) was revealed on the lumbar spine X-ray.

A 52-year-old man with a past medical history of coronary artery disease status post percutaneous coronary intervention with angioplasty 10 years ago, insulin-dependent diabetes mellitus type 2, hyperlipidemia, hypertension, morbid obesity, and a 30-pack-year history of smoking was evaluated for moderate lower back pain progressively worsening over the past few months. The pain prevented him from climbing ladders and stairs required for his job. Physical examination, including vital signs, was normal. His complete blood count was normal. A lumbar spine X-ray showed an incidental extensive, large, fusiform calcified AAA, resembling a football which was 7.4 cm in width and 14.8 cm in length (Figure 1). No other radiological cause for the back pain was evident. The patient underwent an open AAA repair with the graft placement surgery, which was uncomplicated. He was seen two weeks following the surgery in the primary care physician's office, he was fine, and his back pain had resolved. Cigarette smoking, male sex, older age, atherosclerosis, and hypertension are well-known risk factors for AAA development. He was advised to stop smoking.

## Discussion

Calcification of AAA happens due to a chronic inflammatory process, and it has been suggested that the aorta with the aneurysm is more inflamed than the atherosclerotic aorta [8].

According to the US Preventive Services Task Force, one-time screening with ultrasound for AAA is recommended in men aged 65-75 with a history of smoking. One of the studies has demonstrated that two-thirds of acute AAA occurred in patients older than 75 [9]. However, in this case, the patient’s age was significantly lower than the age of screening which may imply screening earlier patients with multiple risk factors. 

Most AAAs are asymptomatic, not detectable on physical examination, and silent until discovered during radiologic testing for other reasons. Symptomatic aneurysms present with back, abdominal, buttock, groin, testicular, or leg pain and require urgent surgical attention [10]. We attributed our patient’s back pain to this massively enlarged aneurysm although there is no way for us to be certain.

There are some studies that investigated whether calcified versus non-calcified AAA impacts the prognosis. Two studies have shown that a lower calcification index was associated with more rapid AAA expansion, implying that calcification might stabilize the aneurysm [11,12]. However, another study suggests that more calcified AAA progresses more rapidly [13]. In one study, the calcium score was significantly related to both all-cause mortality and cardiac mortality (odds ratios (OR) of 2.246 (95% CI 1.591-9.476; p < 0.001) and 1.321 (1.076-2.762; p = 0.003)) respectively [1].

Open versus endovascular treatment has been the subject of debate and a large multinational study demonstrated important similarities in long-term outcomes after endovascular and open aortic repairs across three countries Australia, Germany, and the United States [14]. Another study showed that both surgeries had high mortality, and 68.0% in the endovascular-repair group and 70.0% in the open-repair group died (hazard ratio, 0.96; 95% confidence interval [CI], 0.82 to 1.13). During the first four years of follow-up, overall survival appeared to be higher with endovascular repair than with open repair; from year 4 through year 8, overall survival was higher in the open-repair group; and after eight years, overall survival was once again higher in the endovascular-repair group (hazard ratio for death, 0.94; 95% CI, 0.74 to 1.18). None of these trends were significant. Aneurysm-related deaths were 2.7% in the endovascular-repair group and 3.7% in the open-repair group [15].

## Conclusions

In a high-risk patient, low back pain can be the presenting symptom of large calcified aortic aneurysm and a simple X-ray should be considered for the initial workup of the back and aortic abnormalities. Furthermore, it potentially might be considered as a screening tool to help with early diagnosis and to improve cardiovascular outcomes in younger patients with CAAA. Calcification did not stabilize our patient’s aneurysm since he presented with a very large and highly calcified one. Extensive calcification of the abdominal aorta should be followed more closely and repaired when appropriate.
